# Commentary: Social determinants and the health gap: creating a social movement

**DOI:** 10.1093/ije/dyx182

**Published:** 2017-09-01

**Authors:** Michael Marmot

Before the WHO Commission on Social Determinants of Health (CSDH)^1^^,^^2^ had its meeting in Kobe in 2008, we met the Japanese Prime Minister and other senior government people. One said: ‘We used to think that global health was about disease control; we now recognize that it should also embrace heath systems’—hence universal health coverage. I said that the mission of the CSDH was to offer a third approach, complementary to the first two: action on the social determinants of health, vital for achieving health equity.

To put this third approach on the agenda, I said at the outset of the CSDH (I was the chair) that we wanted to create a social movement, using the best evidence on improving society to advance health equity. I was not at all sure what a social movement looked like, but the signs are promising. For the CSDH, we convened nine knowledge networks involving hundreds of scientists and experts, we engaged with civil society, we talked to governments. Many of these people have become advocates for social determinants of health. They, and many others, are part of our social movement.

In Sweden, for example, a parliamentarian said to me publicly in 2013: ‘Commission reports have a half-life of about six weeks; your report is still being discussed in the Swedish Parliament five years after publication’. That parliamentary discussion led to the setting up of a National Commission under the chairmanship of Olle Lundberg—the author of one of the commentaries here. I am told that there are ten ‘Marmot Reviews’ being conducted in different geographical areas of Sweden.

Sweden is not alone. In England, I was commissioned by the government to conduct a review of social determinants of health and health inequalities which was published in 2010 as the Marmot Review, *Fair Society Healthy Lives.*^3^ Further, commissioned by Szuszanna Jakab, WHO Regional Director for Europe, I led a *European Review of Social Determinants and the Health Divide.*^4^^,^^5^ There has been action in many countries.

A further marker of our social movement: in the ten months after we published the English Marmot Review, *Fair Society Healthy Lives*,^3^ in 2010, my colleagues and I gave approximately 190 invited talks in the UK and globally. There was, and still is, a huge thirst for our approach to reducing health inequalities and promoting health equity, through action on the social determinants of health.

It was to capture the knowledge that was synthesized in these reviews, and evidence of what happened since, that I was prompted to write *The Health Gap: The Challenge of an Unequal World.*^6^ I want the third approach, social determinants of health, on the agenda, and want somewhere that policy makers, students and, dare I hope, interested non-experts can find insights into it. When an ear nose and throat surgeon comes to me, as happened recently, and asks humbly if I will sign five copies of *The Health Gap* because he wants his younger colleagues to read it, as he has, it is a marker that my ambition for the book is being fulfilled.

My consistent message is social justice and evidence-based policies as I seek to engage government and others in action on social determinants of health. A commitment to social justice is important but so too is the evidence. Hence *The Health Gap* drew on the evidence provided, in addition to the CSDH, by the nine groups of experts we assembled for the English review and the thirteen task groups whose evidence underpinned the European review, as well as evidence accumulated apart from those reviews.

Although I had in mind a more general audience, I cannot deny that I would like the experts to get something from it, too. I am, of course, hurt that Mike Savage found the book a ‘tad disappointing’. He made the assumption, incorrectly, that I was trying to write a big social science book in the Piketty Atkinson mode, and that as a sociologist he, Savage, felt that I didn’t quite make it. Had a big social science book been my ambition, I might well have considered some of the interesting things Savage has to say. But I was not writing primarily for academic sociologists who were looking for me to relate my work to theories of inequality. I wanted to put down what I think we know about social determinants of health and show that we know enough to take action, right now.

If I was a tad disappointed by a ‘tad disappointing’ from Mike Savage, the views of another social scientist, Aaron Reeves, help—although Danny Kahneman says that the decrement in happiness from something negative is far greater than the gain from something positive.^7^^,^^8^ Reeves writes of my book:

The book also demonstrates an abiding commitment to public engagement with civil society and policymakers and his work has shifted the academic field in profound ways. *The Health Gap* is also inspiring because it provides multiple examples of people becoming persuaded by the evidence and moving to act; this is a particularly timely reminder in an era of post-fact politics that evidence still matters. Marmot brings urgency and intensity to these issues, passionately arguing that inequalities in health are amenable to change.

I have commented that I have tried to make the transition from finishing every report with ‘more research is needed’ to ‘more action is needed’. We know enough to take action. But, I agree completely with Aaron Reeves’s view that we do need to improve our knowledge base. I agree, too, with his particular diagnoses of what is needed:

This uncertainty will persist unless researchers are able to account for path dependence, unpack the specifics of the treatment, and be sensitive to how interventions shape relationality. Addressing this uncertainty will require careful cross-national work that moves beyond randomised controlled trials and even natural experiments to a political economy approach that offers careful documentation of the trends within and between countries. By persistently reminding his readers of the health gaps within and between societies and by insisting that these are amenable to change, Marmot’s book points toward a deeper engagement with the political economy of health.

Reading Reeves’s sensitive and nuanced account of the gaps in our knowledge, there is a fertile area for much needed research. Young researchers, mature ones too, could do well to read Reeves and take note.

Related to the theme of a political economy of health, Reeves quotes Mackenbach’s scepticism about the success of Britain’s New Labour government in achieving their stated aim of reducing health inequalities in the years of their power, 1997–2010.^9^ A recent paper suggested a different conclusion. Barr, Higgerson and Whitehead examined the gap in life expectancy between the most deprived fifth of local authorities in England and the rest. During the period before the New Labour strategy, the life expectancy gap increased; during the strategy period the life expectancy gap decreased. After 2010, the gap began to increase again as it had before the strategy was implemented.^10^ This widening of inequality post-2010 might provide some explanation for a report we, at the UCL Institute of Health Equity, published, showing that the speed of increase in life expectancy in the UK had slowed. [http://www.instituteofhealthequity.org/resources-reports/marmot-indicators-2017-institute-of-health-equity-briefing/marmot-indicators-briefing-2017-updated.pdf].

We did not want to jump to conclusions as to the causes of the slowing down. Whole-of-life social determinants of health inequalities may contribute. We also pointed to policies of austerity that had led to decreases in spending on social care and health since 2010 at a time when the elderly population was growing, and said that it was urgent to investigate possible links.

Julian Baggini, a philosopher, is a gifted communicator of complex ideas. His masterful book *Freedom Regained* examines the consequences for free will of determinism and materialism in readily understandable form.^11^ He brings the same clarity of thought to the issues I raise of social determinants and health inequalities. I particularly like his distinction, when considering unanswered questions, of empirical and normative questions. But unfinished business is not a reason for inaction, he says:

Marmot’s work has made enough clear to us that there is much we ought to be getting on with in public policy without further debate and controversy. But that does not mean all the implications of the research are completely clear and it is only now a matter of political will. A comprehensive response to his findings requires two things that have not yet been completely worked out. The first is an understanding of how malleable the processes which lead inequalities to cause health inequities are. The second is a holistic conception of the good life which would enable us to say when, if at all, we should sacrifice optimal public health for optimal human flourishing.

I agree that there is much more to do in improving our understanding. I do, though, have answers to two of his questions, albeit incomplete. First, Baggini ponders the extent to which the social hierarchy is an intrinsic feature of human life, and that of non-human primates, and how much is the ‘meaning’ of position in the hierarchy determined by culture and society. I have pondered the same question with Robert Sapolsky, who has spent a lifetime studying hierarchies and their consequences for health in baboons on the African savannah.^12^ Sapolsky and I, comparing human and non-human primates, conclude that hierarchies are more or less universal but their consequences for health vary. Health consequences of where an individual is in the social hierarchy depend greatly on forms of social organization, in both human and non-human societies. As I write in *The Health Gap*, the consequences for health of being low status in Estonia and Hungary are much greater than they are in Sweden and Norway. Being relatively disadvantaged in Sweden and Norway is not a good experience, but it is not as bad for your health as being poor in Estonia.

Baggini’s first set of questions then lead to two types of answer. Let us have social and political action that reduce the big inequalities associated with social hierarchies—Reeves’s political economy of health. The second answer, and much of *The Health Gap* is devoted to this, is to understand the social, material and psychosocial processes by which socioeconomic position translates into worse health. The challenge then is to have sufficient evidence to break the links between relative disadvantage and poor health.

As to the normative question, how much is worth changing to achieve better public health, I don’t pretend to have the complete answer here either. That said, I think there is less of a trade-off than Baggini implies. My view is that a society that promotes human flourishing is one that will have better health. And, the greater the equity in human flourishing, the greater the health equity. I lay this out in *The Health Gap.* It is probably somewhere between an empirical and normative position. I am grateful to Baggini for the distinction.

A different kind of critique, that of T K Sundari Ravindran, I also accept as a constructive contribution to the debate. Were I to write the book again, or to write another, I would want to take her concerns, along with those of Reeves and Baggini, on board. Sundari Ravindran was a member of the Women and Gender Equity Knowledge Network (WGEKN) of the Commission on Social Determinants of Health. Her concern is that I focus too much on socioeconomic determinants with insufficient attention on gender and ethnicity. She is right. My abiding concern has been with the social gradient in health. Classify individuals by some measure of socioeconomic position and, all over the world, we find that the higher the position in the hierarchy the better the health. It is precisely the observation of the social gradient in mortality in the Whitehall Studies^13^ that led to my career-long investigations into social determinants of health inequalities. But that’s not all there is.

In *The Health Gap*, I do make reference to gender and the role of education in empowering women. Sundari Ravindran says this is not enough. Her rationale is persuasive:

Intersectionality-informed approaches demand that we take cognizance of multiple identities because broad categorisation of people along a single axis of social stratification such as class, race or gender may lead to misleading assumptions of within-group homogeneity and to ignoring the diversity as well as stratification. Intersectionality posits that different axes of power and domination may be important in different contexts and over time, and that there need not be a pre-determined pattern.

I think she is right. As she says, it relates not only to gender but to race/ethnicity as well.

I comment in *The Health Gap* that in the USA, the health disadvantage of African Americans compared with Whites can largely be explained by the social determinants that I show are related to socioeconomic disadvantage—the causes of the causes. That is to pay insufficient attention to racism and discrimination—the causes of the causes of the causes. In fact, in our new Commission on Equity and Health Inequalities in the Americas, we are giving emphasis to four cross-cutting themes: gender, ethnicity, equity and human rights. Equity and human rights will run through all our work. Intersectionality calls for considering socioeconomic disadvantage, gender and ethnicity in all the work on social determinants of health.

Nancy Adler and I are on the same page in emphasizing both absolute and relative inequalities. She argues that concern with relative deprivation leads one to the mind and psychosocial processes that link social structural forces to health and health inequalities. To that end she developed a Ladder of Subjective Socioeconomic Status. It is, as would be expected, correlated with objective measures of socioeconomic position, but it has independent predictive power of a variety of health outcomes. The ladder might be one way to look at the kinds of differential ‘downstream’ effects of social determinants to which both Reeves and Sundari Ravindran draw attention. They would argue that we also need to look upstream at how social processes affect groups differently.

One conclusion of Adler’s work is that we can intervene at different levels. As she writes:

Consideration of psychosocial processes together with material conditions should enable more effective policies and programs. While modifying structural factors that generate and maintain socioeconomic inequalities will have the most extensive impact in the long run, structural change is slow and uncertain. Psychosocial interventions that buffer the impact of existing socioeconomic conditions can benefit individuals and populations in the interim, and may potentiate the impact of structural changes as they occur. In brief, no one approach is more important; both structural and individual level approaches are indispensable paths to take to mitigate —and eventually eliminate—the health gap.

Well put.

I have been arguing that those of us concerned with social determinants of health need to be concerned with mental as well as physical illness. People concerned with mental illness, psychiatrists and others, need to be concerned with social determinants of health. Much of my insight on this topic comes from Dinesh Bhugra. When he was President of the Royal College of Psychiatrists, the College produced a volume, *No Health Without Mental Public Health*, that took a social determinants of health approach to preventing mental illness.^14^ That report was a significant milestone for me in getting doctors involved in social determinants of health. When I spent a year as President of the World Medical Association, my mission was to involve national medical associations, medical students and junior doctors in social determinants and health equity. To that end we produced a report *Doctors For Health Equity*, setting out what doctors could do, see [http://www.instituteofhealthequity.org/resources-reports/doctors-for-health-equity-world-medical-association-report/doctors-for-health-equity-wma-full-report-pdf.pdf].

Bhugra’s reminder, that half of psychiatric disorders in adulthood start below the age of 15, reinforces the importance of a life course approach to social determinants of health. Mental illness is also central to the social and geographical overlap between crime and ill-health. As I set out in *The Health Gap*, the prevalence of mental illness among prison inmates is very much higher than in the general population. Bhugra also brings in the much-needed perspective of stigma and discrimination, one aspect of the call by Sundari Ravindran to consider other dimensions of inequality. Bhugra writes:

In the melting pot is the discrimination and stigma people with illness especially mental illness face due to a number of factors. To complicate matters further stigma related to poverty, unemployment, poor housing and overcrowding pathologises people who are not seen like ‘one of us’. Thus discrimination and stigma get further complicated as factors related to race, ethnicity, religion, gender or sexual orientation come into play thereby increasing stigma which then feeds into avoiding seeking help thus setting up a vicious cycle.

I am, of course, pleased that Bhugra, a leading psychiatrist, has the view that social determinants of health have come into the sunlight, and:

Social justice for people with mental illness carries embedded within it concepts of human rights including health rights. The aim of social justice is to strengthen and support institutions be they educational, health, judicial or others which can then help eliminate social discrimination through both education and legal frameworks.

The perspective of social justice is relevant beyond mental illness to the whole field of health inequalities.

Olle Lundberg might well have been responding to Mike Savage when he wrote:

To many of us, it might seem self-evident that health inequalities arise from a multitude of differences in living conditions and life chances, but, as a scientific statement, it is quite bold. This is partly because science usually enquires into the finer details and refrains from painting the bigger picture. But in public health sciences, there is also the tendency to end up in dichotomies: ‘upstream or downstream?’, ‘psycho-social or neo-material?’, ‘structural or behavioural?’ Such dichotomies are at odds with the complex picture Marmot draws; each time Marmot faces such a dichotomy, he rejects it as false. And rightly so—the key to understanding the dynamics of inequality is discovering the complexity of factors and how they interplay.

Lundberg emphasizes:

Both in research and in policy-making, is the need to see the generation of inequalities as a dynamic, multi-causal process. Inequalities grow out of a dynamic interplay of many factors and conditions during the entire life-course. Conditions during early life are crucial. Hence, inequalities of all sorts start within the family. It is, therefore, important to offer good quality pre-schools, in particular to children from homes with fewer resources. However, it does not stop there. Schools can amplify the differences which children bring from home, but can also help level out inequalities. Working life, incomes and a range of other living conditions are important, not just individually, but combined, added, and in constant interplay.

Even in egalitarian, social democratic Sweden they have the challenge of health inequalities. It is from Lundberg and his colleagues that I gratefully received the maxim: do something, do more, do better.

**Conflict of interest:** None declared.
